# 吲哚-3-甲醇对肺癌细胞放射敏感性的EGFR依赖性调节

**DOI:** 10.3779/j.issn.1009-3419.2012.07.01

**Published:** 2012-07-20

**Authors:** 骁 肖, 庆慧 孟, 加英 徐, 旸 焦, M ROSEN Eliot, 赛军 樊

**Affiliations:** 1 215123 苏州，苏州大学医学部放射医学与防护学院 School of Radiation Medicine and Protection, Medical College of Soochow University, Suzhou 215123, China; 2 20007 Washington DC, Lombardi Comprehensive Cancer Center, Georgetown University Lombardi Comprehensive Cancer Center, Georgetown University, Washington DC 20007, USA

**Keywords:** 吲哚-3-甲醇, 肺肿瘤, 放射敏感性, 表皮生长因子受体, Indol-3-carbinol, Lung neoplasms, Radiosensitivity, Epidermal growth factor receptor

## Abstract

**背景与目的:**

吲哚-3-甲醇（indole-3-carbinol, I3C）是十字花科蔬菜中一种主要的有效植物化学物质，且具有防癌和抗癌作用。本研究旨在观察I3C是否影响表皮生长因子受体（epidermal growth factor receptor, EGFR）表达水平不同的肺癌细胞放射敏感性。

**方法:**

采用MTT和克隆形成实验方法分别检测肺癌细胞的生长和存活率；siRNA转染方法降低细胞中EGFR蛋白表达水平；Western blot和RT-PCR法分别测定EGFR蛋白和mRNA的表达。

**结果:**

采用无明显毒副作用的5 μmol/L剂量的I3C预处理明显降低了EGFR表达阳性的人肺腺癌H1975和人肺鳞癌H226细胞对γ-射线照射的放射敏感性，而I3C对EGFR表达阴性的人肺鳞癌NIH-H520细胞的放射敏感性则影响非常小。Western blot结果显示I3C可以增加H1975和H226细胞中EGFR蛋白的表达水平和Y845位点磷酸化水平。EGFR siRNA降低了NIH-H1975细胞中EGFR蛋白的表达，增加了细胞的放射敏感性，并有效地降低和抑制了I3C导致的细胞耐辐射效应。

**结论:**

我们的研究结果首次证实I3C可以通过调节EGFR表达和磷酸化水平从而影响肺癌细胞的放射治疗敏感性，提示EGFR可能是I3C影响肺癌放射治疗敏感性的重要靶蛋白。

## Abstract (en)

**Background and objective:**

Indole-3-carbinol (I3C) is a naturally occurring phytochemical found in cruciferous vegetables. The aim of the present study is to investigate the influence of I3C on radiosensitivity in epidermal growth factor receptor (EGFR)-positive and EGFR-negative lung cancer cell lines.

**Methods:**

Human lung adenocarcinoma NIH-H1975 cells and human lung squamous carcinoma NIH-H226 and NIH-H520 cells were routinely cultured in RPMI-1640. MTT assay and clonogenic assay were used to detect cell growth and survival, respectively. Western blot and RT-PRC assay was employed to detect EGFR protein and mRNA expression.

**Results:**

5 μmol/L of I3C significantly reduced radiosensitivity of EGFR-positive NIH-H1975 and NIH-H226 cells, but failed to affect radiosensitivity of EGFR-negative NIH-H520 cells. Furthermore, I3C caused an increased expression of total EGFR and pEGFR (Y845) protein in NIH-H1975 and NIH-H226 cell lines, but not in NIH-H520 cell line. A reduction of EGFR expression by EGFR-siRNA significantly inhibited I3C-caused radioresistance in NIH-H1975 cells.

**Conclusion:**

Our data presented here for the first time demonstrate that I3C reduces radiosensitivity of lung cancer cells by mediating EGFR expression, indicating that EGFR may be an important target for I3C-mediated radioresistance in lung cancer.

近年来我国肺癌的发病率和死亡率迅速上升，已处恶性肿瘤的首位。虽然不同的治疗方法和技术已不断发展和完善，但肺癌的5年生存率的改善仍然不明显。放射治疗是最常用于肺癌的治疗方法之一，其常在手术前后作为辅助治疗手段应用，在一定程度上可以减少局部复发率、明显改善症状、延长患者的生存期。但是肺癌细胞对放射治疗产生耐辐射效应常常降低了放射治疗的疗效和广泛的应用。影响肺癌放射治疗疗效的因素除了放射治疗方法、肺癌临床分期和病理类型、患者免疫状态等因素外^[1]^，药物和饮食也同样可以影响放射治疗的疗效。本实验研究了十字花科蔬菜中研究最多和最重要的一种抗肿瘤成分吲哚-3-甲醇（indole-3-carbinol, I3C）对肺癌细胞放射敏感性的影响及其与表皮生长因子受体（epidermal growth factor receptor, EGFR）的调节关系。

## 1 材料与方法

### 1.1 肺癌细胞培养及试剂

3种肺癌细胞系来自美国Georgetown大学组织和细胞实验中心。培养基为含10%小牛血清的RPMI-1640（Life Technology, USA）。吲哚-3-甲醇（I3C）购置于美国Sigma公司，并溶解在纯度大于99%的二甲基亚砜（DMSO）中，配制成20 mmol/L的储存液存于-20 ℃备用。

### 1.2 细胞照射

将指数生长的不同肺癌细胞分为I3C+γ-射线照射组和DMSO+γ-射线照射组，前者为肺癌细胞在5 μmol/L I3C作用24 h后，后者为肺癌细胞在同量的DMSO（作为对照）作用24 h后，在室温下进行γ-射线照射。照射采用^137^Cs γ-射线照射源进行，剂量率为0.85 Gy/min。

### 1.3 MTT方法

取对数生长期的细胞，酶消化，接种于96孔培养皿中，24 h后细胞密度大约为40%-50%。除去培养液，加已加入了5 μmol/L I3C的培养液至相应的细胞孔中。置培养箱中继续培养24 h和48 h后加入MTT（5 g/L）20 μL/孔，孵育4 h，弃液后加入DMSO 100 μL/孔，振荡。最后采用酶标仪在570 nm波长检测每孔细胞OD值，采用细胞存活率=加药组吸光度值/对照组吸光度值×100%的方式计算细胞存活率。

### 1.4 克隆形成实验

取指数生长期的肺癌细胞，经酶消化，计数后并按不同照射剂量（0 Gy-8 Gy）在60 mm直径的培养皿中接种不同数量的细胞数，γ-射线照射组和I3C+γ-射线照射组每个剂量设6个副孔。培养贴壁8 h后，实验组加含不同剂量I3C至相应的培养孔，对照组只换培养液，等药物处理24 h后分别进行0 Gy-8 Gy γ-射线照射，照射后重新换培养基继续培养大约2周至克隆形成。然后结晶紫染液染色，冲洗和晾干，显微镜下观察并计数细胞数大于50个的细胞克隆数。按照“0 Gy剂量集落数/细胞接种数×100%”公式计算0 Gy组克隆形成效率，并按照某一剂量照射组的集落数/该组细胞接种数×未照射组克隆形成率（survival fraction, SF）。

### 1.5 siRNA转染

EGFR siRNA（sc-29301）和Control siRNA（sc-37007）购置于美国Santa Cruz生物技术公司。取对数生长期的NIH-H1975细胞，调整细胞密度，接种至12孔培养皿，24 h后除去培养液，采用无血清和抗生素的培养液清洗。并根据Lipofectamine 2000转染试剂的操作指南（Invitrogen, USA）将siRNA与Lipofectamine 2000分别用适量无血清和抗生素的培养液稀释和混匀，20 min室温静置后取出并直接加入培养孔中，置于培养箱培养72 h进行I3C处理和/或γ-射线照射。

### 1.6 Western blot方法

细胞采用胰酶消化，离心5 min（2, 500 r/min），弃上清，加入IP裂解液转至1.5 mL离心管并置于冰上裂解2 h。4 ℃离心5 min（13, 000 r/min）后，取上清，并采用BCA法检测样品中蛋白的含量。取总蛋白100 μg混合蛋白上样缓冲液，在100 ℃加热5 min，并采用10%的SDS-PAGE进行电泳分离，电泳后转至PVDF膜上。转膜结束后，采用5%脱脂奶粉封闭1 h。加入抗EGFR单克隆抗体（sc-7134）或抗EGFR磷酸化（phospho Y845）抗体（ab5636）4 ℃过夜，洗膜后加入相对应的二抗室温孵育2 h，洗膜，最后加ECL显色试剂盒显影，胶片洗涤和干燥。测量目的条带的光密度值。另外，采用抗β-actin多克隆抗体（I-19）检测β-actin蛋白表达作为样品内参以及转膜的效果。抗EGFR、抗β-actin抗体和二抗都购置于美国Santa Cruz生物技术公司。抗EGFR磷酸化（phospho Y845）抗体购置于美国Abcam公司。

### 1.7 RT-PCR方法

根据RNA抽提操作手册采用Trizol试剂提取组织总RNA。提取的RNA经电游鉴定其完整性，并采用紫外分光光度计测定260 nm与280 nm吸光度值从而计算RNA的纯度与浓度。EGFR上游引物为：5′-TGG AGC TAC GGG GTG ACC GT-3′，下游引物为5′-TGG AGC TAC GGG GTG ACC GT-3′；GAPDH mRNA上游引物为：5′-AAG CCC ATC ACC ATC TTC-CAG-3′，下游引物5′-AGG GGC CAT CCA CAG TCT TCT-3′。按试剂说明操作完成cDNA的合成，反应体系为：dNTP Mixture 1 μL，Template RNA 1 μg，RNase Free dH_2_O加至到10 μL，在65 ℃孵育5 min后，直接加入5×Prime Script Buffer 4 μL、RNase Inhibitor 0.5 μL、Prime Script RTase 0.5 μL和RNase Free dH_2_O 5 μL，然后分别置于30 ℃ 10 min、42 ℃ 20 min以及95 ℃ 5 min。取逆转录反应液2 μL，加入10×PCR Buffer 2 μL、dNTP Mixture 0.8 μL、上下游引物各1 μL和RNase Free dH_2_O 13 μL。反应条件为：94 ℃预变性5 min，94 ℃变性1 min，60 ℃退火30 s，72 ℃延伸1 min，35个循环后在72 ℃延伸7 min。取最终反应产物10 μL在2%琼脂糖凝胶进行电泳，溴乙啶染色，最后在紫外光下照相记录。

### 1.8 统计学方法

所有实验均重复3次，取平均值，结果用Mean±SD表示。采用SPSS 19.0统计软件对相关数据进行统计分析，组间比较采用*t*检验，以*P* < 0.05为差异有统计学意义。

## 2 结果

### 2.1 I3C对肺癌细胞放射敏感性的影响

首先我们采用MTT分析法比较了5.0 μmol/L I3C的24 h处理对NIH-H1975、NIH-H226和NIH-H520三种不同肺癌细胞系生长和增殖的影响。正如图 1A所示，5.0 μmol/L I3C对三种肺癌细胞生长影响较小，三种肺癌细胞对I3C的敏感性并无明显差异，且抑制率均 < 15%，表明5.0 μmol/L I3C的24 h处理并不产生明显的细胞毒毒副作用。接下来，采用5.0 μmol/L I3C预处理不同细胞24 h，然后细胞接受不同剂量的γ-射线照射，并完成细胞克隆形成实验。从图 1B可见，三种肺癌细胞放射敏感性分别为NIH-H520 > NIH-H1975 > NIH-H226，抑制50%细胞生长所需的辐射剂量（IC_50_）分别为0.4 μmol/L、1.0 μmol/L和1.4 μmol/L，而抑制90%细胞生长所需的辐射剂量（IC_90_）则分别为2.7 μmol/L、3.9 μmol/L和4.3 μmol/L。IC_50_和IC_90_在各细胞系之间相比较差异都具有统计学意义（*P* < 0.05）。另外，与对照DMSO+γ-射线照射的细胞存活曲线相比，I3C+γ-射线照射的NIH-H1975和NIH-H226细胞存活曲线明显上移，即放射敏感性在不同程度上降低。例如，与DMSO+γ-射线照射细胞的IC_50_（1.0 μmol/L）和IC_90_（3.9 μmol/L）相比，I3C+γ-射线照射的NIH-H1975细胞的IC_50_和IC_90_分别为4.0 μmol/L和7.0 μmol/L，相比差异都具有统计学意义（*P* < 0.05）。但是，5 μmol/L I3C的24 h预处理并未明显改变照射后NIH-H520细胞的存活曲线（图 1B）。

**1:**
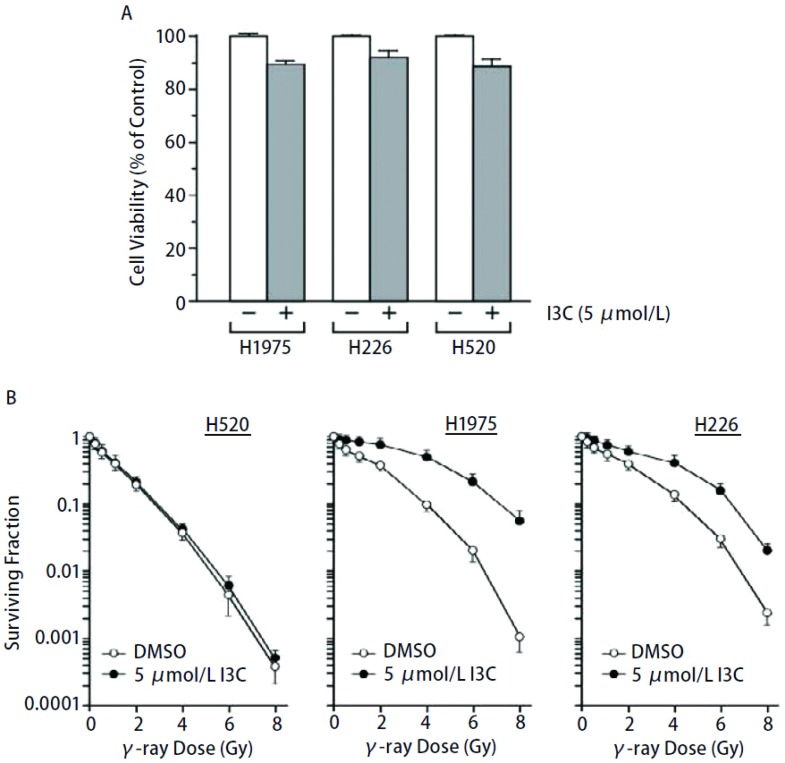
I3C对肺癌细胞放射敏感性的影响。A：5 μmol/L I3C 24 h处理对三种肺癌细胞生长的影响；B：照射前5 μmol/L I3C的24 h预处理对三种肺癌细胞放射敏感性的影响。 Effect of I3C on radiosensitivity of lung cancer cells. A: Effect of I3C (5 μmol/L, 24 h) on growth of three lung cancer cell lines; B: Effect of I3C at 5 μmol/L (24 h) before irradiation on radiosensitivity of three lung cancer cell lines.

### 2.2 I3C对肺癌细胞株放射敏感性影响与EGFR表达水平的关系

已知肺癌细胞株中EGFR表达水平与肺癌放射敏感性有密切的相关性^[2-4]^。EGFR表达水平的差异和改变是否在I3C调节肺癌细胞放射敏感性中起到一定作用?为此，我们采用Western blot方法比较了NIH-H1975、NIH-H226和NIH-H520三种肺癌细胞系中内源性EGFR蛋白的表达水平。如图 2所示，在NIH-H1975和NIH-H226细胞中可以不同程度地检测到EGFR蛋白的表达，NIH-H226细胞中的EGFR蛋白表达水平略比NIH-H1975细胞高。但在NIH-H520肺癌细胞中并未检测到EGFR蛋白的表达。这些EGFR表达水平结果与以前已报道的结果完全一致^[5]^。

**2:**
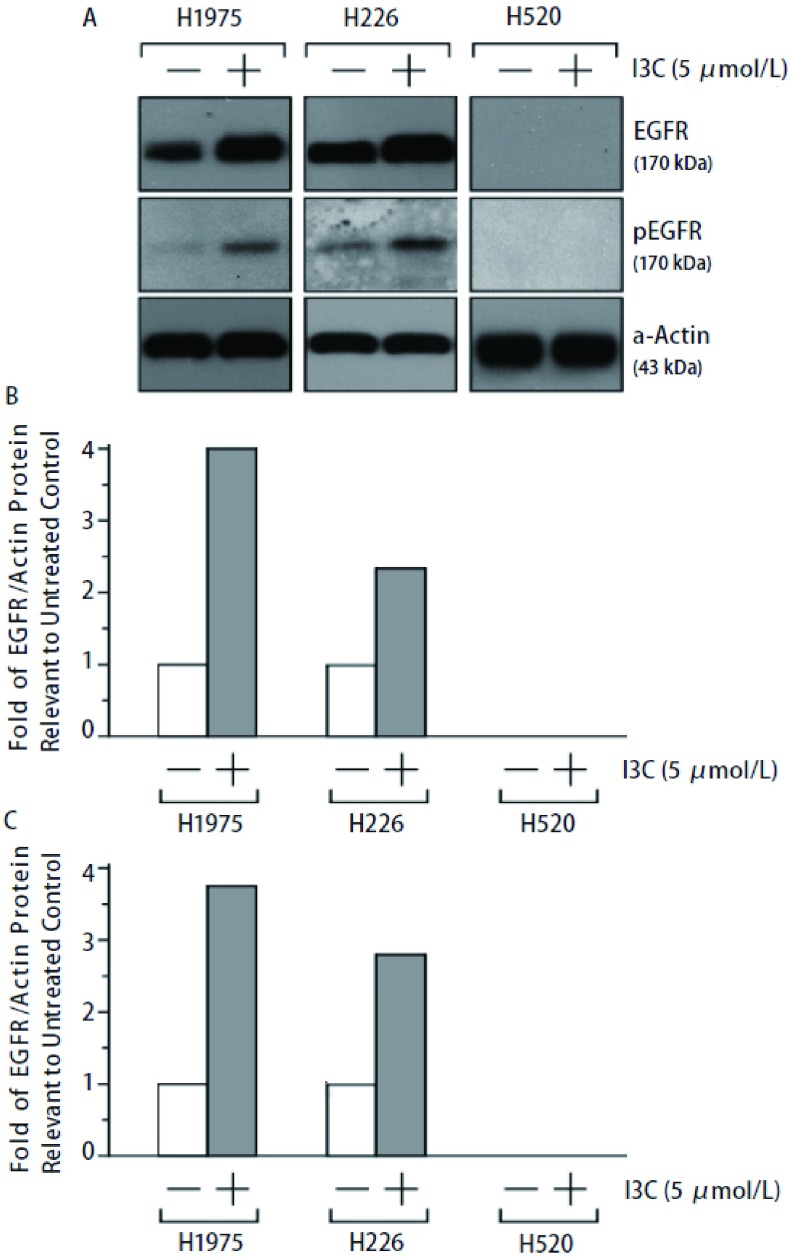
5 μmol/L I3C 24 h处理对三种肺癌细胞EGFR蛋白表达和ERFR磷酸化（Y845）水平的影响。A：EGFR及pEGFR蛋白表达情况；B：三种细胞中EGFR的表达情况；C：三种细胞中pEGFR表达情况。 Effect of I3C (5 μmol/L, 24 h) on expression of EGFR protein and phospho-EGFR (Y845) in three lung cancer cell lines. EGFR: epidermal growth factor receptor. A: the expression levels of EGFR and pEGFR; B: the expression level of EGFR in three lung cancer cell lines; C: the expression level of pEGFR in three lung cancer cell lines.

正如我们预期的，I3C处理的NIH-H520细胞中的EGFR蛋白无任何变化，依然无法检测到EGFR蛋白的表达（图 2）。I3C处理不同程度地增加了NIH-H1975和NIH-H226两种肺癌细胞系中的EGFR蛋白表达水平，且I3C增加NIH-H1975细胞中EGFR表达量（4.1倍）比其增加NIH-H226细胞中EGFR量（2.3倍）要高。另外，I3C也在不同程度上提高了NIH-H1975细胞（3.7倍）和NIH-H226细胞（2.8倍）中的EGFR蛋白在Y845位点的磷酸化水平。

为了进一步了解EGFR表达水平改变在I3C调节肺癌细胞放射敏感性中可能的作用，我们采用脂质体介导EGFR siRNA转染至NIH-H1975细胞中降低EGFR的表达水平以观察其放射敏感性的变化。如图 3所示，与NIH-H1975母细胞（Control）相比，对照siRNA（control siRNA）转染细胞中的EGFR蛋白（图 3A）和mRNA（图 3B）表达水平无任何明显改变。但转染了EGFR siRNA的NIH-H1975细胞中EGFR蛋白和mRNA的表达水平都出现明显下降，EGFR蛋白下降至母细胞EGFR量的8%。这些结果表明EGFR siRNA的转染能有效地抑制EGFR蛋白和mRNA的表达水平。同时，我们也比较了I3C预处理对NIH-H1975母细胞、对照siRNA和EGFR siRNA转染细胞放射敏感性的影响。如图 3C所示，照射后NIH-H1975母细胞和对照siRNA转染的细胞存活曲线比较没有明显差异，而且I3C也明显导致了NIH-H1975母细胞和对照siRNA转染细胞的放射敏感性降低，降低的程度两者之间也无明显差异。但是，虽然EGFR siRNA明显提高了细胞的放射敏感性，而I3C导致的耐辐射现象则明显减弱。

**3:**
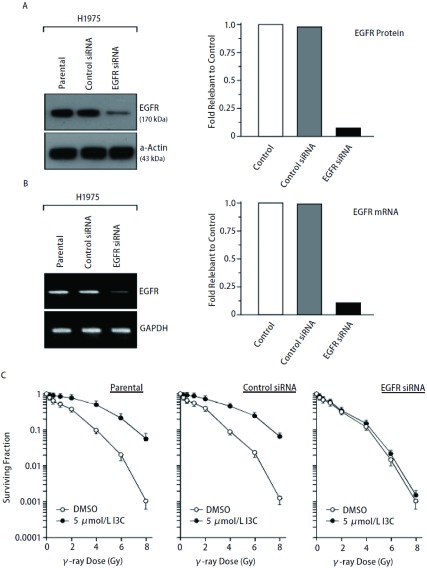
降低EGFR表达对I3C调节放射敏感性的影响。EGFR siRNA转染对H1925细胞中EGFR蛋白(A) and mRNA (B)表达的影响；C：照射前5 μmol/L I3C的24 h预处理对H1925母细胞，Control siRNA和EGFR siRNA转染的H1925细胞放射敏感性的影响。 Effect of EGFR decrease on radiosensitivity. Effect of EGFR siRNA transfection on expression of EGFR protein (A) and mRNA (B) in H1925 cells; C: Effect of I3C with 24 h treatment of 5 μmol/L before irradiation on radiosensitivity of H1925 parental cells and H1925 cells transfected with Control siRNA and EGFR siRNA.

## 3 讨论

EGFR是一种细胞膜表面的糖蛋白受体，具有酪氨酸激酶活性，是原癌基因*C-erb1*（HER-1）的表达产物，属于表皮生长因子家族（erbB家族）。EGFR在包括肺癌在内许多肿瘤组织中都呈现高水平表达，与其配体结合后被活化，在肿瘤细胞的增殖、转移和肿瘤血管的生成中起到重要的作用，故EGFR已成为肿瘤治疗的重要靶点，包括吉非替尼、厄洛替尼等针对EGFR靶点的特异性酪氨酸激酶抑制剂也已进入临床治疗治疗试验中^[6, 7]^。当肿瘤细胞受到电离辐射照射后，EGFR进入细胞核，参与并加快了DNA损伤的修复，从而降低了放射损伤^[8]^。研究^[3]^表明EGFR高表达的肿瘤细胞对放射治疗表现为比较低的放射敏感性。另外，通过采用特异性EGFR抑制剂抑制EGFR的表达及其调节通路的活性，都可以通过促进肿瘤细胞凋亡、改变辐射诱导的细胞周期阻滞，增加辐射诱导的DNA损伤修复能力等作用机制而降低肺癌的放射治疗敏感性^[3, 8-14]^。而且研究^[14]^也发现EGFR蛋白结构中酪氨酸激酶域、L858R和DeltaE746-E750三个蛋白区域是EGFR调节放射敏感性的关键所在。

我们的实验研究结果表明，①三种肺癌细胞中EGFR表达水平与其放射敏感性成负相关，EGFR蛋白表达水平最高的NIH-H226细胞，其放射敏感性则最低。相反，EGFR表达阴性的NIH-H520细胞则表现高放射敏感性。不同肺癌细胞的放射敏感性与其内源性EGFR蛋白表达水平成负相关，通过EGFR siRNA降低EGFR表达水平可以提高肺癌细胞的放射敏感性；②辐射照射前给予I3C预处理可以明显增加EGFR阳性的肺癌NIH-H1975和NIH-H226细胞放射敏感性；③I3C可以增加EGFR阳性的肺癌细胞中EGFR蛋白的表达以及在EGFR蛋白Y845位点的磷酸化水平。在NIH-H1975细胞中，I3C可提高EGFR蛋白表达量和Y845位点的磷酸化水平，降低细胞的放射敏感性程度更明显。而对EGFR阴性的肺癌NIH-H520细胞放射敏感性则无明显影响。这些实验研究结果表明I3C导致肺癌细胞放射敏感性降低的原因可能是与其增加EGFR表达和磷酸化有关。其四，采用EGFR siRNA降低肺癌细胞中EGFR蛋白表达水平可以降低I3C导致的耐辐射抗性。一系列体外实验研究结果都证实I3C处理可以降低肺癌细胞放射敏感性，EGFR蛋白表达及其磷酸化水平的增加可能是I3C导致的肺癌细胞耐辐射抗性的主要原因。这些研究结果说明降低EGFR表达水平可以增加NIH-H1975细胞的放射敏感性或起着关键性作用。换句话说，EGFR可能是I3C调节肺癌细胞放射敏感性的靶蛋白。虽然目前EGFR信号传导的具体机制还不清楚，但研究发现EGFR激活后可激活不同下游信号通路，比较明确的主要有ras-raf-MEK-erk/MAPK信号通路和P13K-PKC-IKK信号通路两条途径。因此，我们实验室目前正在深入了解EGFR调节的这两条下游信号通路的激活是否在I3C耐辐射抗性中起到关键作用，同时也正在研究与放射敏感性相关的EGFR蛋白结构中酪氨酸激酶域、L858R和DeltaE746-E750三个蛋白区域是否也参入I3C导致的耐辐射效应。另外，EGFR不同蛋白位点的磷酸化是EGFR行使其生物学功能的重要机制。虽然我们已证实I3C可以诱导EGFR蛋白Y845位点的磷酸化，但对EGFR蛋白其它磷酸化位点，例如Y1068、Y1173和Y1148，是否同样有激活效应也是我们需要进一步研究的内容。同样，是否这些磷酸位点的磷酸化改变在I3C调节肺癌放射敏感性中起到关键和必要的作用将为我们深入研究I3C导致耐辐射效应提供新的研究方向。

I3C是小白菜、菜心、大白菜、芥蓝、青花菜、叶芥菜等十字花科蔬菜中主要的植物化学物质，其具有的防癌和抗癌作用引起研究人员和临床肿瘤医生的极大的关注和广泛的研究^[15, 16]^。世界上十字花科植物有3, 200多种，来源非常广，是我国饮食中常见的一类蔬菜。运输冷冻、切碎和烹调等食物加工处理和咀嚼可使十字花科菜中的细胞受到破坏，导致葡糖异硫氰酸盐经黑芥子硫苷酶水解后可以生成I3C及异硫氰酸盐。I3C进入胃后在胃酸环境中非常不稳定，通过缩合反应形成其它的多聚物，包括3, 3’-二吲哚甲烷（DIM）、吲哚[3, 4-b]并咔唑（ICZ）和2-（吲哚-3-基甲基）-3, 3二吲哚甲烷（LTr-1）等。并且，在血液中也是以这几种代谢产物为主要成分存在。这些吲哚-3-甲醇的酸性缩合物也都具有非常强的生物活性，其中以DIM和ICZ两种产物活性最强。因此，我们将非常有兴趣地了解是否I3C的这些代谢产物也同样具有影响肺癌细胞放射敏感性的功效。吲哚-3-甲醇是十字花科蔬菜中主要的活性成分，因此肺癌放射治疗患者在临床放射治疗前如果食用一定量的十字花科蔬菜或摄取I3C后是否将对其放射治疗的疗效产生一定的影响目前还需要做进一步探讨和研究。

另外，我们早期的研究结果揭示，I3C是DNA损伤修复基因乳腺癌易感基因-1（breast cancer susceptibility gene 1, BRCA1）的诱导剂，可以明显地增加肿瘤细胞中BRCA1基因的表达水平^[17, 18]^。近年来，研究^[19-24]^表明BRCA1表达可能也是肺癌一重要的独立或辅助预后指标。但引人关注的是越来越多的国内外研究^[25-35]^结果已证实BRCA1是参与包括吉西他滨、多烯紫杉醇、顺铂等在内的肺癌常用化疗药物敏感性调节和预后的相关基因。例如在顺铂化疗耐药肺癌患者癌组织样本中的BRCA1蛋白阳性表达率明显高于化疗敏感患者样本。也就是说，BRCA1表达阴性有利于肺癌化疗治疗疗效，BRCA1的高表达则降低了肺癌细胞对化疗的反应或治疗效果^[26-35]^。因此，BRCA1是预测肺癌一定化疗药物疗效的重要指标。而且，结合EGFR和BRCA1的指标更能有效地指导肺癌的个体化化疗治疗^[26, 36]^。但至目前为止，国内外还没有任何有关BRCA1调节肺癌放射敏感性的相关文献报道。因此，除了研究BRCA1基因表达水平自身是否与肺癌放射治疗疗效有关外，BRCA1的表达改变是否也参与在I3C的EGFR依赖性的肺癌细胞放射敏感性调节机制中也有待进一步深入研究。
